# The R.B.N.A. and Mental Nurses

**Published:** 1897-04-17

**Authors:** 


### THE R.B.N.A. AND MENTAL NURSES.

Dr. S. Duncan Greenlees, Medical Superintendent of the Grahamstown Asylum, South Africa, writes: I have watched with great interest the discussion on the proposed amalgamation of asylum workers with the R.B.N.A., both in your pages and in the *British Medical Journal*. As one who carried on the systematic training of nurses in an English asylum before the Medico-Psychological Association introduced its certificate, and at a time when the majority of medical superintendents offered much opposition to any form of training, I view with pleasure the progress this work is making, and regret that the R.B.N.A. should refuse to welcome to its ranks a band of conscientious, hard-working, and well-trained men and women such as are now to be found in most modern asylums. The opposition offered by some members of the R.B.N.A. to this scheme of union can result in nothing but damage to their own cause. It is surely to their interest to increase their numbers by several thousands, and if their objection rests on the plea that asylum nurses know nothing of general nursing, then the latter can retaliate by pointing out that the hospital nurse knows nothing of mental nursing. The two should go together, or the Association should be made only to comprise hospital nurses, and so we might found the Royal British Hospital Nurses' Association and the Royal British Asylum Nurses' Association. But this would not be good policy. "Union is strength," and when we speak of a nurse we are quite at liberty to call her either an asylum or hospital nurse. If, on the other hand, this opposition is to continue, then I would advise all asylum workers to band themselves together, and there are many thousands who are ready to do so, and form a R.B.A.N.A., to distinguish themselves from the R.B.H.N.A., who are totally incapable of nursing a mental case. A new association has just been formed calling itself "Asylum Helpers," consisting of asylum nurses who have passed the Medico-Psychological Association nursing examination. If they could change the name it would do more to raise the status of the asylum nurse and be a move in the direction of amalgamation. A good deal of slight has been put on the examination instituted by the Medico-Psychological Association, but I know of several instances of hospital nurses who, after two years' excellent training, were unable to answer either the physiological questions or those on general nursing submitted in the

Army Medical Library
15 8765
Washington, D. C. U. S. A.

November examination paper. This fact speaks for itself, and shows that the authorities are particularly anxious that all holding the certificate for mental nursing issued by the Medico-Psychological Association should show their competency for undertaking the nursing of an ordinary case—whether it be fever or fracture, as well as the nursing of simply mental cases. In this country I have striven for years to induce the Medical Council to recognise our certificate, and to register "mental nurses," but hitherto I have been unsuccessful—most of their time being devoted to licensing midwives to practice and the discussion of medical ethics. Opposition to the progress of mental nursing, and to raising the status of the mental nurse—for this is what it comes to, and is the result of jealousy—will, in these days, but defeat its own object. While mental nursing is progressive hospital nursing is stationary, and it would be well for the former to help the latter. Otherwise the time may come when the hospital nurse will be glad to throw in her lot with the asylum nurse. I think in a matter of this kind there should be a sort of "give and take" policy adopted. Why should not the hospital nurse qualify for mental nursing and the asylum nurse qualify for general nursing. The time devoted would not be lost, and the public, whom we are all anxious to serve, would then know that when a nurse was engaged—no matter what the disease was—reliance could be placed upon her capacity to nurse the case through to a successful termination. If this were done then one association of nurses is all that is required ; if it is not done a division in the ranks will ensue and prove of unfortunate detriment to the cause of nursing generally.

